# Modified Mediterranean Diet Score and Cardiovascular Risk in a North American Working Population

**DOI:** 10.1371/journal.pone.0087539

**Published:** 2014-02-04

**Authors:** Justin Yang, Andrea Farioli, Maria Korre, Stefanos N. Kales

**Affiliations:** 1 Department of Environmental Health, Environmental & Occupational Medicine & Epidemiology, Harvard School of Public Health, Boston, Massachusetts, United States of America; 2 The Cambridge Health Alliance, Harvard Medical School, Cambridge, Massachusetts, United States of America; 3 Department of Medical and Surgical Sciences, University of Bologna, Bologna, Italy; College of Pharmacy, University of Florida, United States of America

## Abstract

**Introduction:**

Greater adherence to a Mediterranean diet is linked to lower risk for cardiovascular morbidity/mortality in studies of Mediterranean cohorts, older subjects, and/or those with existing health conditions. No studies have examined the effects of this dietary pattern in younger working populations in the United States. We investigated the effects of Mediterranean diet adherence on cardiovascular disease (CVD) biomarkers, metabolic syndrome and body composition in an occupationally active, non-Mediterranean cohort.

**Methods:**

A cross-sectional study in a cohort of 780 career male firefighters, ages 18 years or older, from the United States Midwest. No dietary intervention was performed. A modified Mediterranean diet score (mMDS) was developed for assessment of adherence to a Mediterranean dietary pattern from a previously administered life-style questionnaire that examined pre-existing dietary habits. Clinical data from fire department medical examinations were extracted and analyzed.

**Results:**

Obese subjects had significantly lower mMDS, and they reported greater fast/take-out food consumption (p<0.001) and intake of sweetened drinks during meals (p = 0.002). After multivariate adjustment, higher mMDS was inversely related to risk of weight gain over the past 5 years (odds ratio [OR]: 0.57, 95% confidence interval [CI]: 0.39–0.84, p for trend across score quartiles: 0.01); as well as the presence of metabolic syndrome components (OR: 0.65, 95% CI: 0.44–0.94, p for trend across score quartiles: 0.04). Higher HDL-cholesterol (p = 0.008) and lower LDL-cholesterol (p = 0.04) were observed in those with higher mMDS in linear regression after multivariate adjustment for age, BMI and physical activity.

**Conclusions:**

In a cohort of young and active US adults, greater adherence to a Mediterranean-style dietary pattern had significant inverse associations with metabolic syndrome, LDL-cholesterol and reported weight gain, and was significantly and independently associated with higher HDL-cholesterol. Our results support the potential effectiveness of this diet in young, non-Mediterranean working cohorts, and justify future intervention studies.

## Introduction

Lifestyle behaviors have long been correlated with lowering cardiovascular disease (CVD) risk. [1] In particular, the Mediterranean diet has consistently been associated with better health status, decreased all-cause mortality and protective/ameliorative effects on chronic diseases. [2]–[7] Specifically, this type of diet is associated with benefits regarding cardiovascular risk factors such as obesity, hypertension, diabetes mellitus and metabolic syndrome [3], [8]–[11], as well as on the relative risks of CVD-related morbidity and mortality [2], [9], [12]–[14].

The Mediterranean diet is collection of similar eating habits traditionally followed in at least 16 countries bordering the Mediterranean Sea. [15] It is characterized by high consumption of olive oil, fruits, vegetables, non-refined breads and cereals, potatoes, legumes and nuts; moderate consumption of fish and poultry; a low intake of dairy products, red meat, processed meats and sweets; and moderate wine intake with meals. [6], [12], [15].

Although several studies have measured Mediterranean diet adherence with a scoring system and have reported inverse associations with CVD morbidity and mortality, those investigations were primarily conducted on older subjects, those with existing health conditions and/or among Mediterranean populations. [2], [4], [6], [7], [12], [15]–[17] Little is known about the effects of Mediterranean-style diet among young working groups in non-Mediterranean countries. To the best of our knowledge, no studies have examined this dietary pattern in a North American occupational cohort. We investigated a modified Mediterranean diet score (mMDS) to assess Mediterranean diet adherence and its associations in a population of United States (US) Midwestern firefighters. No intervention was performed. Rather, we investigated the association between cardiovascular risk markers and the extent to which the firefighters’ reported dietary habits conformed to a Mediterranean diet pattern using the mMDS.

## Subjects and Methods

### Study Population and Study Design

We conducted a cross-sectional analysis within an ongoing longitudinal study of a young, occupationally active cohort of career male firefighters. The participants were age 18 years or older from 11 fire departments in two Midwestern states. Mediterranean diet adherence was assessed from responses to a life-style questionnaire, and CVD biomarkers were measured during the firefighters’ baseline medical evaluations. The cohort and data collection have been described in detail elsewhere. [18], [19] Inclusion criteria for the mMDS investigation were: 1) completion of a fire department-sponsored medical examination including a maximal exercise test; 2) completion of the life-style questionnaire; 3) absence of work-restrictions at examination; and 4) signed informed consent.

### Ethics Statement

The study was approved by the Institutional Review Board (IRB) of the Harvard School of Public Health and by local IRBs (Chesapeake IRB and National Development and Research Institute [NDRI] IRB).

### Assessment of Adherence to Mediterranean Diet

**Figure 1:**
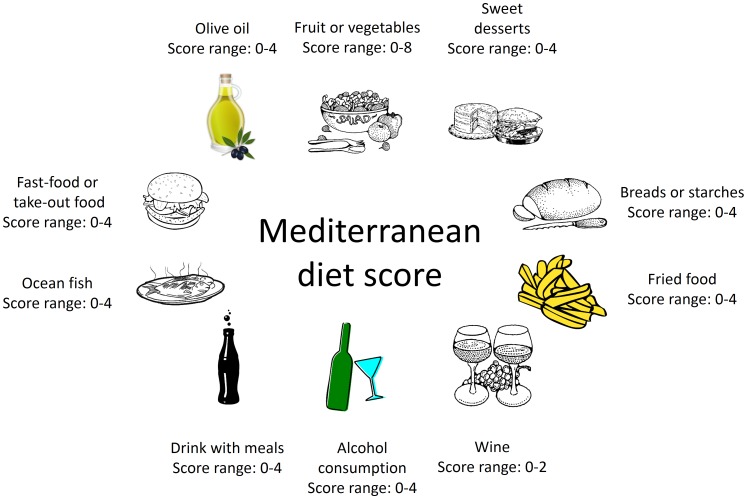
Mediterranean diet food domains and ranges of modified Mediterranean diet item scores (mMDS) among different categories.

A modified Mediterranean diet score (mMDS) was developed by examining questions from our existing life-style questionnaire for relevance to Mediterranean diet components and adherence to traditional Mediterranean eating patterns based on previous studies. [4], [6], [12], [13] To construct the scoring system, we identified fifteen question areas including the following food domains: frequency of consuming fast/take-out food; weekly serving(s) of fruit and vegetables; frequency of sweet dessert consumption; cooking oil/fat use (olive oil versus others); weekly fried food consumption; type of breads/starches eaten with meals (refined versus whole grain); frequency of consuming ocean fish; and beverage consumption which included wine/alcohol drinking frequency and type of drink(s) consumed with most meals. **Figure 1** shows the generalized Mediterranean diet component categories and score ranges we developed using our questionnaire.

For each Mediterranean diet-associated question, a 4-point scale was developed where a score of 4 is given to the response that best represents a Mediterranean-style diet, and 0 is assigned to the choice that least conforms to a Mediterranean-style diet. A detailed description of how question responses were coded and mMDS scores were attributed for each item is provided in **Table S1 and Method S1**. For questions on “drinks with most meals” we assigned scores for different types of beverages based on likely calorie intake and antioxidant components per serving. In a working population, meals at the workplace and at home may differ substantially. Hence, we surveyed separately cooking oil use, breads/starches and beverages at home and at work, and then weighted each item’s consumption by the proportion of weekly meals each participant reported that he consumed at the workplace or at home, respectively. We scored overall alcohol intake because moderate ethanol consumption is consistently associated with a reduced risk of CVD outcomes. [20] Furthermore, additional points for wine intake were also scored separately because authorities consider wine to be an integral part of Mediterranean diet. [21]–[23] We then obtained each individual’s final total mMDS by summing the scores across all items. The total mMDS score has a possible range of 0 (no conformity to a Mediterranean-style diet) to 42 (maximal conformity to a Mediterranean-style diet based on our scoring system).

### Assessment of CVD Risk Factors and Covariates

Detailed descriptions of the collection of anthropometric, clinical and laboratory data from this cohort are summarized in previous studies. [24], [25] Briefly, body mass index (BMI, kg/m^2^) was recorded for all study subjects from measured height and weight. Body fat (%) was estimated by Bioelectrical Impedance Analyzer (BIA) or with skin fold measures, and was added to the medical evaluation protocol while the study was in progress. Cardio-respiratory fitness was measured using symptom- limited maximal treadmill exercise testing with estimation of oxygen consumption (metabolic equivalents [METS]) according to the Bruce protocol. The prevalence of metabolic syndrome and its individual components among the study population were determined using modified criteria from the Joint Scientific Statement. [26], [27].

Reports of weight change over the last 5 years and physical activity were extracted from the lifestyle questionnaire. The following question was used to assess weight change: “In the last 5 years, my body weight has gone…”. Possible answers included: down a lot (>10 pounds [>4.54 kg]); down a little (5–10 pounds [2.27–4.54 kg]); not changed (<5 pounds [<2.27 kg]); up a little (5–10 pounds [2.27–4.54 kg]); and up a lot (>10 pounds [>4.54 kg]). Weekly physical activity was estimated from average reported exercise frequency and the average reported duration of aerobic/cardio sessions each week. The product of these two responses yielded the average duration of total weekly aerobic exercise expressed in minutes. [19].

### Statistical Analysis

Statistical analyses were carried out using Stata 12.1 SE (Stata Corp, College Station, TX, US). Trends across ordered groups were analyzed with the Cuzick nonparametric test (continuous variables) or with a score test for linear trend of the log odds (dichotomous variable). Differences in the mean distribution of continuous variables were tested with univariate and multivariate analysis of variance. Linear regression models were fitted to study the effect of a unitary increase in mMDS. We explored the distribution of continuous variables by plotting histograms. We then transformed the following right-skewed variables (METS, triglycerides, total cholesterol, LDL-cholesterol, HDL-cholesterol, total cholesterol/HDL, and blood sugar) by taking their natural logarithm. Finally, we assessed the normality assumption for log-transformed variables by applying the skewness and kurtosis test for normality [28]. We fitted ordered logistic regression models to study naturally ordered dependent variables (i.e. metabolic syndrome score and weight change over the past five years). Parallel regression assumption was tested via the Brant test. A P value of less than 0.05 (two-sided) was considered statistically significant.

## Results

**Figure 2:**
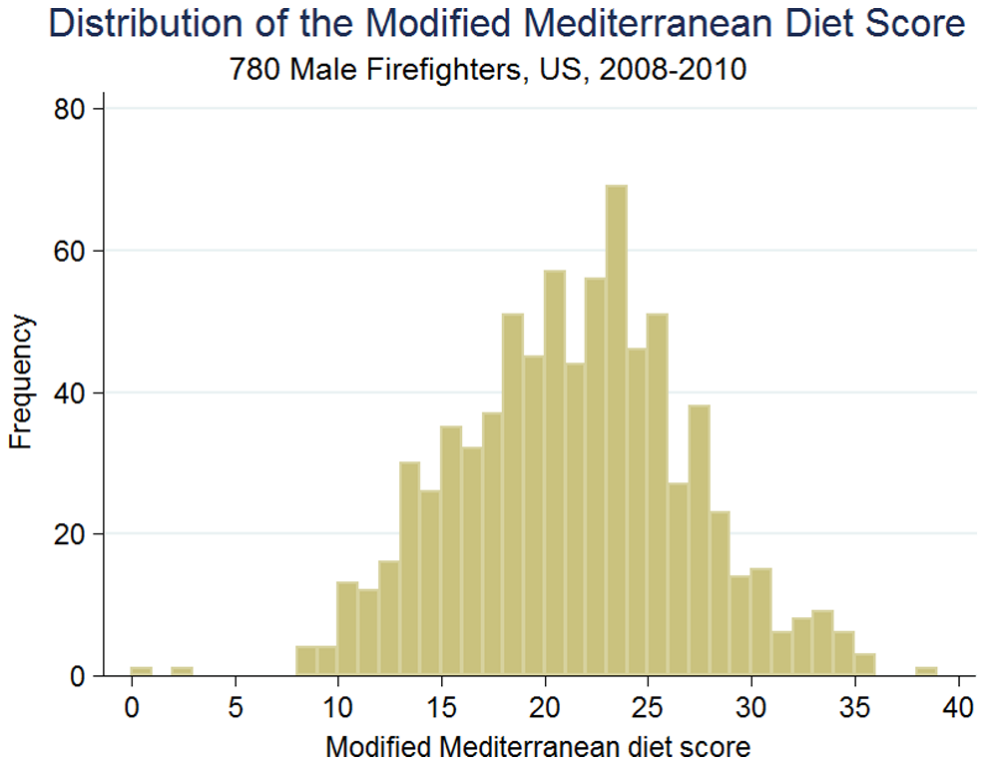
Distribution of the modified Mediterranean diet score (mMDS) among 780 male subjects.

In this study, 780 (97% of the total eligible database) male firefighters met the inclusion criteria and were selected for the main analyses. Twenty-six subjects were excluded due to incomplete information. In **Table 1**, we present the distribution of personal characteristics by stratifying our study population into four BMI categories. The mean mMDS in the study population was 21.3 (SD 5.6) (**Figure 2**). Normal weight subjects had significantly higher mMDS than obese firefighters (p for trend 0.008). Beverages taken with meals, both at home and at work, as well as the frequency of eating fast/take-out food were significant determinants for the observed differences in mMDS across BMI categories.

**Table 1:** Descriptive results of characteristics and modified Mediterranean diet scores (mMDS) among 780 subjects.

	Normal weight		Overweight		Obese class I		Obese class II/III		
	*(18.5≤BMI≤24.9)*		*(25.0≤BMI≤29.9)*		*(30.0≤BMI≤34.9)*		*(BMI≥35.0)*		
Characteristics and mMDS results	(N = 108)		(N = 401)		(N = 203)		(N = 85)		*P* trend
Age, mean (SD)	35.6	(10.0)	37.2	(8.4)	38.9	(8.0)	38.6	(8.1)	<0.001
Body fat percentage, mean (SD)^2^	16.5	(7.0)	21.2	(4.2)	27.5	(3.0)	32.1	(3.9)	<0.001
Max. METS achieved during ETT, mean (SD)	13.7	(1.6)	13.1	(1.5)	12.2	(1.8)	10.9	(2.0)	<0.001
Percentage of max. HR achieved during ETT, mean (SD)	99.0	(5.2)	98.8	(6.2)	97.5	(5.7)	96.5	(7.1)	0.001
Total weekly aerobic exercise (min*wk^-1^), median (IQR)	86	(26–131)	79	(56–131)	79	(56–131)	56	(11–124)	0.003
Current smoker, n (%)	19	(17.6)	80	(20.0)	39	(19.2)	19	(22.1)	0.563
Number of meals at the firehouse, median (IQR)	6	(3–6)	6	(3–6)	6	(3–6)	6	(4.5–7.5)	0.018
Single-item mMDS									
-Fast/take-out food consumption, mean (SD)	2.8	(1.2)	2.7	(1.1)	2.6	(1.1)	2.3	(1.2)	<0.001
-Fruit and vegetable consumption^3^, mean (SD)	2.2	(1.8)	2.3	(1.9)	2.1	(1.7)	2.0	(1.7)	0.109
-Sweet desserts consumption, mean (SD)	2.6	(1.2)	2.7	(1.2)	2.8	(1.1)	2.8	(1.2)	0.389
-Primary cooking oil/fat use at home, mean (SD)	2.2	(1.6)	2.6	(1.5)	2.4	(1.5)	2.6	(1.6)	0.391
-Secondary cooking oil/fat used at home, mean (SD)	1.8	(1.6)	1.8	(1.5)	1.9	(1.5)	1.9	(1.5)	0.239
-Primary cooking oil/fat use at work, mean (SD)	1.9	(1.4)	2.0	(1.3)	2.1	(1.4)	2.2	(1.3)	0.088
-Secondary cooking oil/fat used at work, mean (SD)	1.7	(1.7)	1.7	(1.6)	1.8	(1.5)	1.7	(1.6)	0.952
-Fried food consumption, mean (SD)	2.2	(0.9)	2.2	(0.9)	2.2	(0.9)	2.0	(1.0)	0.146
-Breads/starches consumed at home, mean (SD)	2.6	(1.9)	2.6	(1.9)	2.6	(1.8)	2.3	(1.9)	0.647
-Breads/starches consumed at work, mean (SD)	1.7	(1.7)	1.5	(1.7)	1.8	(1.8)	1.8	(1.8)	0.358
-Ocean fish consumption, mean (SD)	1.6	(0.9)	1.5	(0.7)	1.5	(0.8)	1.5	(0.7)	0.829
-Drinks taken with meals at home, mean (SD)	2.9	(1.6)	2.6	(1.7)	2.3	(1.7)	2.1	(1.7)	<0.001
-Drinks taken with meals at work, mean (SD)	2.7	(1.7)	2.6	(1.7)	2.5	(1.6)	1.9	(1.8)	0.002
-Quantity of alcoholic beverages, mean (SD)	2.1	(1.6)	2.1	(1.5)	2.0	(1.5)	1.8	(1.6)	0.093
-Wine consumption, mean (SD)	0.1	(0.4)	0.1	(0.5)	0.2	(0.5)	0.1	(0.5)	0.667
Total mMDS, mean (SD)	21.7	(5.5)	21.6	(5.4)	21.0	(5.7)	19.8	(5.6)	0.008

1 Nonparametric test for trend (Cuzick) performed for all columns except current smokers, where score test for trend was performed.

2 Information available for 244 subjects.

3 Score doubled due to combining two different domains.

Abbreviations: BMI, body mass index (kg/m^2^); ETT, exercise treadmill test; IQR, interquartile range; max, maximum; METS, metabolic equivalents; SD, standard deviation.

In **Table 2**, we show CVD risk factors stratified by mMDS quartiles. After adjusting for age and BMI, significant inverse associations with mMDS were observed for body fat percentage (p = 0.0179), triglycerides (p = 0.0463), and total cholesterol to high-density lipoprotein-cholesterol (HDL-c) ratio (TC/HDL-c, p<0.0001), while HDL-c (p = 0.0001) and METS (p = 0.0047) were positively associated with mMDS. When further adjusted by physical activity, body fat percentage, HDL-c, and TC/HDL-c remained significantly associated with mMDS.

**Table 2:** Statistical analyses of anthropometric and metabolic variables associated with cardiovascular disease risk by quartiles of modified Mediterranean diet score (mMDS) in 780 male subjects.

		I quartile≤17.5(N = 194)	II quartile17.6–21.4(N = 195)	III quartile21.5–25.0(N = 190)	IV quartile≥25.0(N = 201)	P value (analysis of variance)				
Risk factor	N	*Mean (SD)*	*Mean (SD)*	*Mean (SD)*	*Mean (SD)*	*Unadjusted*	*Adjusted by age*	*Adjusted by age and BMI*	*Adjusted by age, BMI and physical activity*	
Age^1^ (years)	780	38.2 (8.6)	37.0 (8.2)	37.9 (9.2)	37.1 (8.4)	0.4195		0.5448	0.5877	
BMI^1^	780	29.9 (4.7)	29.4 (4.6)	29.2 (4.2)	28.7 (4.2)	0.0756	0.0950		0.6369	
Body fat^1^ (%)	233	25.2 (6,8)	24.4 (7.0)	22.7 (5.7)	22.2 (6.3)	0.0327	0.0372	0.0179	0.0281	
Resting SBP^1^ (mmHg)	780	122.4 (12.6)	121.8 (12.7)	122.7 (11.8)	122.8 (13.3)	0.8546	0.8606	0.6013	0.9156	
Resting DBP^1^ (mmHg)	780	80.4 (8.5)	79.7 (7.6)	79.2 (7.8)	79.9 (8.2)	0.5749	0.5812	0.6125	0.6219	
Resting HR^1^ (bpm)	780	71.5 (11.6)	68.9 (11.2)	69.5 (11.3)	68.2 (11.5)	0.0220	0.0316	0.0887	0.4126	
Percentage of max. HR achieved during ETT^1^	765	98.0 (6.5)	98.4 (6.2)	98.5 (6.3)	98.1 (5.3)	0.7963	0.7920	0.7771	0.7796	
METS^2^	766	12.3 (1.2)	12.7 (1.1)	12.7 (1.1)	13.0 (1.1)	0.0002	0.0003	0.0047	0.3784	
Triglycerides^2,3^ (mg/dL)	780	140.4 (1.8)	125.8 (1.7)	122.8 (1.7)	115.8 (1.8)	0.0065	0.0093	0.0463	0.4604	
Total cholesterol^2,3^ (mg/dL)	780	196.5 (1.3)	191.0 (1.2)	191.4 (1.2)	186.3 (1.2)	0.0567	0.0926	0.1348	0.3275	
LDL-cholesterol^2,3^ (mg/dL)	759	120.3 (1.3)	117.0 (1.4)	115.0 (1.4)	110.2 (1.3)	0.0328	0.0427	0.0599	0.0982	
HDL-cholesterol^2,3^ (mg/dL)	780	41.7 (1.3)	43.9 (1.3)	44.2 (1.3)	46.6 (1.3)	0.0001	0.0001	0.0009	0.0258	
Total cholesterol/HDL^2,3^	780	4.7 (1.4)	4.4 (1.3)	4.3 (1.4)	4.0 (1.3)	<0.0001	<0.0001	<0.0001	0.0035	
Blood sugar^2,3^ (mg/dL)	780	93.2 (1.2)	92.6 (1.2)	92.9 (1.2)	91.1 (1.2)	0.5690	0.6896	<0.8387	0.9289	

1 Arithmetic mean and standard deviation.

2 Geometric mean and standard deviation.

3 Analysis of variance performed using log-transformed dependent variable.

Abbreviations: BMI, body mass index; DBP, diastolic blood pressure; bpm, beats per minute; ETT, exercise treadmill test; HDL, high-density lipoprotein; HR, heart rate; LDL, low-density lipoprotein; METS, metabolic equivalents; SD, standard deviation, SBP, systolic blood pressure.

In **Table 3**, we present fully adjusted linear regression models of CVD risk factors and mMDS. For every unit increase of mMDS, we observed a decrease of 0.4% in the geometric mean of low-density lipoprotein-cholesterol (LDL-c), an increase of 0.4% in the geometric mean of HDL-c, and a 0.7% decrease in the TC/HDL-c. Maximal METS achieved were also positively associated with compliance to the Mediterranean diet (0.2% increase per unit increase in mMDS).

**Table 3:** Effect of a unitary increase in the modified Mediterranean diet score (mMDS) on anthropometric and metabolic variables related to cardiovascular disease risk and in 780 male subjects. B coefficient from linear regression models^1^.

		Linear regression models											
		*Unadjusted*			*Adjusted by age*			*Adjusted by age and BMI*			*Adjusted by age, BMI and physical activity*		
Dependent variable	*Mean (SD)*	β	Change	*P*	β	Change	*P*	β	Change	*P*	β	Change	*P*
BMI^2,5^	29.3 (4.4)	−0.087	−0.3%	0.002	−0.082	−0.3%	0.004				−0.051	−0.2%	0.093
Body fat^2,5^ (%)	23.6 (6.6)	−0.248	−1.1%	0.002	−0.216	−0.9%	0.006	−0.075	−0.3%	0.121	−0.042	−0.2%	0.404
Resting SBP^2,5^ (mmHg)	122.3 (12.7)	0.041	0.0%	0.612	0.061	0.0%	0.452	0.113	+0.1%	0.156	0.055	0.0%	0.515
Resting DBP^2,5^ (mmHg)	80.0 (8.1)	−0.047	−0.1%	0.368	−0.036	0.0%	0.494	0.008	0.0%	0.874	0.007	0.0%	0.899
Resting HR^2,5^ (bpm)	69.4 (11.5)	−0.259	−0.4%	<0.001	−0.246	−0.4%	0.001	−0.200	−0.3%	0.006	−0.107	−0.2%	0.157
% Maximum HR during ETT^2,5^	98.3 (6.1)	0.002	0%	0.957	0.004	0.0%	0.926	−0.010	0.0%	0.806	0.011	0.0%	0.796
METS^3,4,6^	12.7 (1.1)	0.005	+0.5%	<0.001	0.004	+0.4%	<0.001	0.003	+0.3%	<0.001	0.002	+0.2%	0.028
Triglycerides^3,4,6^ (mg/dL)	125.1 (1.8)	−0.013	−1.3%	<0.001	−0.013	−1.3%	<0.001	−0.010	−1.0%	0.005	−0.005	−0.5%	0.189
Total cholesterol^3,4,6^ (mg/dL)	191.1 (1.2)	−0.004	−0.4%	0.003	−0.003	−0.3%	0.007	−0.003	−0.3%	0.014	−0.003	−0.3%	0.055
LDL-cholesterol^3,4,6^ (mg/dL)	115.6 (1.4)	−0.006	−0.6%	0.004	−0.005	−0.5%	0.010	−0.005	−0.5%	0.018	−0.004	−0.4%	0.040
HDL-cholesterol^3,4,6^ (mg/dL)	44.1 (1.3)	0.007	+0.7%	<0.001	0.007	+0.7%	<0.001	0.006	+0.6%	<0.001	0.004	+0.4%	0.008
Total cholesterol/HDL^3,4,6^	4.3 (1.4)	−0.011	−1.1%	<0.001	−0.011	−1.1%	<0.001	−0.009	−0.9%	<0.001	−0.007	−0.7%	0.001
Blood sugar^3,4,6^ (mg/dL)	92.4 (1.2)	−0.001	−0.1%	0.310	−0.001	−0.1%	0.544	0.000	0.0%	0.926	0.000	0.0%	0.672

1 Number of subjects with complete data: 780, except for body fat % (n = 233), % maximum HR during ETT (n = 765), METS (n = 766) and LDL-cholesterol (n = 759).

2 Arithmetic mean and standard deviation.

3 Geometric mean and standard deviation.

4 Linear regression model conducted on a log-transformed dependent variable.

5 Percent change in the arithmetic mean of the dependent variable per unitary increase in the Mediterranean diet score.

6 Percent change in the geometric mean of the dependent variable per unitary increase in the Mediterranean diet score.

Abbreviations: BMI, body mass index; DBP, diastolic blood pressure; bpm, beats per minute; ETT, exercise treadmill test; HDL, high-density lipoprotein; HR, heart rate; LDL, low-density lipoprotein; METS, metabolic equivalents; SD, standard deviation, SBP, systolic blood pressure.

The distribution of metabolic syndrome score also varied by mMDS (**Table 4**). In ordered logistic regression analysis, subjects in the highest quartile of mMDS had a 35% lower risk of a one unit increase in metabolic syndrome score (Odds ratio [OR]: 0.65 after adjusted for age and physical activity, 95% confidence interval [CI]: 0.44–0.94, p for trend across mMDS quartiles: 0.039).

**Table 4:** Associations of 1) metabolic syndrome score and 2) reported body weight change to modified Mediterranean Diet Score (mMDS) in 780 male subjects by estimates from ordered logistic regression models.

METABOLIC SYNDROME SCORE 1																			
	0	1		2		3		4		5	Unadjusted estimates			Estimates adjusted by age			Estimates adjusted by age and physical activity		
mMDS quartiles^2^	*N*	*N*		*N*		*N*		*N*		*N*									
	*(%)*	*(%)*		*(%)*		*(%)*		*(%)*		*(%)*	*OR*	*(95% CI)*	*P*	*OR*	*(95% CI)*	*P*	*OR*	*(95% CI)*	*P*
I	26	54		49		33		22		10	1.00	Ref.		1.00	Ref.		1.00	Ref.	
	(13.4)	(27.8)		(25.3)		(17.0)		(11.3)		(5.2)									
II	46	52		41		35		19		2	0.67	(0.47–0.94)	0.023	0.71	(0.50–1.00)	0.052	0.75	(0.53–1.07)	0.108
	(23.6)	(26.7)		(21.0)		(18.0)		(9.7)		(1.0)									
III	44	50		41		32		15		8	0.70	(0.49–0.99)	0.044	0.72	(0.50–1.03)	0.070	0.80	(0.56–1.15)	0.238
	(23.2)	(26.3)		(21.6)		(16.8)		(7.9)		(4.2)									
IV	59	59		32		35		10		6	0.50	(0.35–0.71)	<0.001	0.52	(0.36–0.74)	<0.001	0.65	(0.44–0.94)	0.021
	(29.4)	(29.4)		(15.9)		(17.4)		(5.0)		(3.0)									
*P trend*													<0.001			0.001			0.039
**REPORTED WEIGHT CHANGE IN THE LAST 5 YEARS**																			
	**Down >10 lbs.**		**Down 5–10 lbs.**		**Stable**		**Up 5–10 lbs.**		**Up>10 lbs.**		**Unadjusted** **estimates**			**Estimates adjusted by** **age and BMI**			**Estimates adjusted by** **age, BMI and** **physical activity**		
**mMDS quartiles** 2	***N***		***N***		***N***		***N***		***N***										
	***(%)***		***(%)***		***(%)***		***(%)***		***(%)***		***OR***	***(95% CI)***	***P***	***OR***	***(95% CI)***	***P***	***OR***	***(95% CI)***	***P***
I	9		19		59		61		42		1.00	Ref.		1.00	Ref.		1.00	Ref.	
	(4.7)		(10.0)		(31.1)		(32.1)		(22.1)										
II	15		16		66		68		24		0.73	(0.51–1.05)	0.036	0.73	(0.51–1.05)	0.089	0.78	(0.54–1.13)	0.194
	(7.9)		(8.5)		(34.9)		(36.0)		(12.7)										
III	12		31		53		65		28		0.70	(0.48–1.01)	0.039	0.72	(0.50–1.05)	0.087	0.83	(0.57–1.21)	0.338
	(6.4)		(16.4)		(28.0)		(34.4)		(14.8)										
IV	31		20		78		54		17		0.43	(0.30–0.62)	<0.001	0.44	(0.31–0.65)	<0.001	0.57	(0.39–0.84)	0.005
	(15.5)		(10.0)		(39.0)		(27.0)		(8.5)										
*P trend*													<0.001			<0.001			0.010

1 Determined adding one point for each of the following: obesity (BMI≥30 kg/m^2^); reduced HDL-cholesterol (<40 mg/dL); hypertriglyceridemia (≥150 mg/dL); elevated blood pressure (systolic ≥130 mmHg or diastolic ≥85 mmHg) or antihypertensive drug treatment; or hyperglycemia (blood glucose ≥100 mg/dL).

2 mMDS quartiles definitions: I quartile: total mMDS score ≤17.5, II quartile: total mMDS score between 17.6–21.4, III quartile: total mMDS between 21.5–25.0, IV quartile: total mMDS ≥25.0.

NB Brant test was used to explore violations of the proportional odds assumption.

Abbreviations: 95%CI, 95% confidence intervals; OR, odds ratio.

Subjects with a high mMDS were also less likely to report weight gain over the last 5 years. Using ordered logistic regression, participants in the highest quartile of mMDS showed a significantly reduced odds of weight gain (OR adjusted by age, BMI and physical activity: 0.57, 95%CI: 0.39–0.84, p for trend across mMDS quartiles: 0.01).

## Discussion

This study provides comprehensive evidence of statistically significant beneficial associations between higher mMDS and CVD risk factors among a young and occupationally active North American cohort. Subjects who were obese had a significantly lower mMDS score. This difference was primarily because obese participants were more inclined to have sweetened drinks or beverages with less nutritional value during meals, and they were more likely to consume fast/take-out foods. We observed higher HDL-c and lower LDL-c in those with better mMDS, which persisted after multivariable adjustment. Furthermore, metabolic syndrome score was inversely associated with Mediterranean-style diet in our study. We also observed a consistent beneficial trend in reported weight gain over the past 5 years among those with lower mMDS, which remained significant after multivariate adjustment. Because we examined the associations of adherence to a Mediterranean dietary pattern based on pre-existing habits without any intervention, our study likely underestimates the benefits of a traditional, fully compliant Mediterranean diet. In other words, the effects of an intervention study that educated the participants and prescribed a specific Mediterranean diet might be expected to be even greater.

HDL-c and LDL-c are well-established independent risk factors for CVD. [29] Although previous research has observed positive changes in lipid profiles in groups adhering to Mediterranean diet, those studies were primarily conducted in older subjects, those with pre-existing conditions, and/or Mediterranean cohorts; and most of them did not observe significant findings in HDL-c after covariate adjustment. [3], [9], [16], [17], [30] In our cohort, we observed significantly higher HDL-c and lower LDL-c in those with greater mMDS, in both multivariate analyses of variance and liner regression models after covariate adjustment. Our study is the first to observe these relationships with Mediterranean-style diet in a group of young working adults in the U.S.

The potential protective effect of a higher mMDS on metabolic syndrome, which has been well correlated with increased CHD and overall mortality [31], was another significant finding in this study. Participants in the highest quartile of mMDS compared to the lowest quartile had a 35% lower risk for the presence of an additional metabolic syndrome component after adjustment for age and physical activity. Therefore, our results suggest adherence to a Mediterranean-pattern diet in a young and active cohort could potentially reduce CVD-risk clustering and metabolic syndrome prevalence.

A trend in reduction of total mMDS associated with obesity was also observed. This finding is in agreement with previous studies suggesting subjects with better adherence to Mediterranean diet were less likely to be obese. [32]–[34] In this study, we expanded this inverse relation with the observation of a consistent trend in reported weight gain over the past 5 years among those with lower mMDS even after multivariate adjustment that included physical activity. We also observed significantly higher maximal METS achieved in the entire cohort, as well as lower body fat with higher mMDS in the subgroup of 233 participants who had this assessed. Therefore, we hypothesize that adherence to the Mediterranean diet can positively influence fitness and body composition.

Our study also revealed interesting findings regarding beverage consumption. The intake of sweetened beverages, which are not traditionally part of a Mediterranean diet, are well known to be correlated with obesity and increased cardiovascular risk. [1], [35]–[37] Sugary drinks are considered the greatest contributor to added-sugar intake in the U.S. [36], [38], [39] Therefore, we believe sweetened beverage consumption is an important dietary determinant and should be incorporated into the Mediterranean diet scoring systems. Additionally, contrary to patterns observed in traditional Mediterranean countries, we observed very low wine consumption. This was likely the result of socio-cultural preferences, where 60% of our participants reported beer as their drink-of-choice. Educating existing drinkers in similar groups of workers on avoiding alcoholic beverages lacking important antioxidant properties might be an area of interest.

The finding that subjects with lower body fat/BMI and higher physical activity level had lower scores in fast/take-out food consumption further agrees with studies that associated fast-food consumption with obesity [40]–[42] and cardiometabolic risk. [43] While consumption of fast/take-out foods is very prevalent among the U.S. working population, [44], [45] further research on educating employees about healthier food and introducing Mediterranean-style choices at work in different occupational cohorts could be a way to curtail the current obesity epidemic in the U.S. [46], [47].

Our study has several limitations. First, our life-style questionnaire was originally designed to obtain general dietary information, rather than assess a specific diet pattern. Therefore, information on total energy intake and certain traditional Mediterranean food domains (e.g. nuts and legumes) were not collected and accounted for in the analyses. However, we believe that these two food groups are not highly consumed in the population studied and therefore, would not have influenced scores very much. We were also limited in our ability to assess the associations of ocean fish consumption by the very low consumption observed in our cohort likely due to its geographic setting in U.S. Midwest. Similarly, only a small proportion of our study population were regular wine drinkers. Thus, we had limited statistical power to study the possible beneficial effects of moderate wine consumption. Nonetheless, our questionnaire did address the majority of the essential components of a traditional Mediterranean diet. Additionally, our survey more accurately reflected dietary patterns in a working U.S. population with additional categories tailored to “American” eating habits inconsistent with a Mediterranean diet, as well as questions that assessed potential differences for consumption patterns at work compared to in the home. As our participants were only informed that the study was related to heart disease, they were not aware that Mediterranean diet or any other specific diet was of interest to the overall study. This fact likely decreased reporting bias; although we cannot completely rule out bias based on widespread popular knowledge of more and less “heart healthy” foods. [32].

To the best of our knowledge, this study is the first to assess Mediterranean-style diet adherence and CVD risk factors in a young, working cohort in the U.S. The main strength of our study is the homogeneous population that minimized confounding factors such as gender or socioeconomic differences (e.g., educational level, income or occupation). Also, the study of dietary patterns within a well-defined occupational group allows indirect control for job-related psychosocial factors, which are known to be determinants of eating awareness. [48] Another strength is our data collection procedures: anthropometric, clinical and laboratory data were collected using standardized procedures, and the biological plausibility of the observed relationships across different CVD risk parameters in this cohort has been verified in previous studies of physical activity, obesity and physical fitness. [25], [26], [49] Thus, it is very unlikely that our findings are due to chance or bias.

In conclusion, in a cohort of young working North American male adults, metabolic syndrome score, LDL-cholesterol and reported weight gain had significant inverse associations with increasing mMDS, while higher HDL-cholesterol was found to be significantly and independently associated with higher mMDS. The observed relationships support the potential effectiveness of a Mediterranean-style diet in younger, working cohorts in non-Mediterranean countries, and justify future intervention studies.

## Supporting Information

Table S1 **Questions extracted from the life-style questionnaire that constructed the modified Mediterranean diet score (mMDS) system.**
(DOC)

Method S1 **Calculation of the modified Mediterranean diet score (mMDS).**
(DOC)
