# Supplementary material for: Modified Mediterranean Diet Score and Cardiovascular Risk in a North American Working Population
Source: PLoS One. 2014 Feb 4;9(2):e87539. doi: 10.1371/journal.pone.0087539 (PMC3913651; doi:10.1371/journal.pone.0087539)
Supplement: Table S1 — Questions extracted from the life-style questionnaire that constructed the modified Mediterranean diet score (mMDS) system. (DOC) [file pone.0087539.s001.doc]

**Table S1.** Questions extracted from the life-style questionnaire that constructed the modified Mediterranean diet score (mMDS) system. For each question below, the upper row indicates choices given on the questionnaire and the lower row indicates mMDS score given to each answers.

| A) Most weeks, I eat fast-food or take-out food… a week | 1 or less | | | | | | 2-4 | | | | | | 5-7 | | | | | | 8-10 | | | | | | 11 or more | | | | | | Missing | | | | | |
| --- | --- | --- | --- | --- | --- | --- | --- | --- | --- | --- | --- | --- | --- | --- | --- | --- | --- | --- | --- | --- | --- | --- | --- | --- | --- | --- | --- | --- | --- | --- | --- | --- | --- | --- | --- | --- |
|  | 4 | | | | | | 2 | | | | | | 1 | | | | | | 0 | | | | | | 0 | | | | | | 0 | | | | | |
| B) Servings of fruit and vegetables | 0 | | | | 1 or less | | | | | | 2-3 | | | | | 3-4 | | | | | | 5-6 | | | | | 7 or more | | | | | | Missing | | | |
|  | 0 | | | | 0 | | | | | | 2 | | | | | 4 | | | | | | 6 | | | | | 8 | | | | | | 0 | | | |
| C) Most weeks, I eat sweet desserts… | Never | | | | 1 or less | | | | | | 2-3 | | | | | 3-4 | | | | | | 5-6 | | | | | Every day | | | | | | Missing | | | |
|  | 4 | | | | 4 | | | | | | 3 | | | | | 2 | | | | | | 1 | | | | | 0 | | | | | | 0 | | | |
| D) At my house, the oil or fat that we used more often…^a^ | Butter | | | Lard or other animal fat | | | | | | Margarine | | | | Corn or vegetable oil | | | | | Benechol or Smart Balance | | | | | Olive oil | | | | Other | | | | | | Missing | | |
|  | 0 | | | 0 | | | | | | 1 | | | | 2 | | | | | 3 | | | | | 4 | | | | 0 | | | | | | 0 | | |
| E) At my house, the oil or fat that we used second most often…^1^ | Butter | | | Lard or other animal fat | | | | | | Margarine | | | | Corn or vegetable oil | | | | | Benechol or Smart Balance | | | | | Olive oil | | | | Other | | | | | | Missing | | |
|  | 0 | | | 0 | | | | | | 0.5 | | | | 1 | | | | | 1.5 | | | | | 2 | | | | 0 | | | | | | 0 | | |
| F) At the firehouse, the oil or fat that we used more often…^2^ | Butter | | | Lard or other animal fat | | | | | | Margarine | | | | Corn or vegetable oil | | | | | Benechol or Smart Balance | | | | | Olive oil | | | | Other | | | | | | Missing | | |
|  | 0 | | | 0 | | | | | | 1 | | | | 2 | | | | | 3 | | | | | 4 | | | | 0 | | | | | | 0 | | |
| G) At the firehouse, the oil or fat that we used second most often…^2^ | Butter | | | Lard or other animal fat | | | | | | Margarine | | | | Corn or vegetable oil | | | | | Benechol or Smart Balance | | | | | Olive oil | | | | Other | | | | | | Missing | | |
|  | 0 | | | 0 | | | | | | 0.5 | | | | 1 | | | | | 1.5 | | | | | 2 | | | | 0 | | | | | | 0 | | |
| H) Most weeks, I eat fried foods… | Never | | | | 1 or less | | | | | | 2-3 | | | | | 3-4 | | | | | | 5-6 | | | | | Every day | | | | | | Missing | | | |
|  | 4 | | | | 3 | | | | | | 2 | | | | | 1 | | | | | | 0 | | | | | 0 | | | | | | 0 | | | |
| I) At my house, we mostly eat as breads or starches with meals ^1^ | White bread/macaroni/pasta/ rice or potatoes | | | | | | | | | French/Italian/other crusty bread | | | | | | | | | Whole wheat/multi-grain bread/pasta or brown rice | | | | | | | | | Missing | | | | | | | | |
|  | 0 | | | | | | | | | 2 | | | | | | | | | 4 | | | | | | | | | 0 | | | | | | | | |
| J) At the firehouse, we mostly eat as breads or starches with meals ^2^ | White bread/macaroni/pasta/ rice or potatoes | | | | | | | | | French/Italian/other crusty bread | | | | | | | | | Whole wheat/multi-grain bread/pasta or brown rice | | | | | | | | | Missing | | | | | | | | |
|  | 0 | | | | | | | | | 2 | | | | | | | | | 4 | | | | | | | | | 0 | | | | | | | | |
| K) Most weeks I eat baked, boiled, grilled or blackened (not fried) ocean fish | Never | | | | 1 or less | | | | | | 2-3 | | | | | 3-4 | | | | | | 5-6 | | | | | Every day | | | | | | Missing | | | |
|  | 0 | | | | 1 | | | | | | 2 | | | | | 3 | | | | | | 4 | | | | | 4 | | | | | | 0 | | | |
| L) Most weeks, I drink … alcoholic beverages | 0 | 1-2 | | | | 3-4 | | | 5-6 | | | 7-8 | | | 9-10 | | | 11-12 | | 13-14 | | | 15-16 | | | 17-18 | | | 19-20 | | | 21+ | | | | Missing |
|  | 0 | 2 | | | | 2 | | | 4 | | | 4 | | | 4 | | | 4 | | 4 | | | 4 | | | 4 | | | 4 | | | 1 | | | | 0 |
| M) When I drink alcohol, I mostly drink… | White wine | | | | | | Red Wine | | | | | | Beer | | | | | | Hard liquors | | | | | | Don’t drink | | | | | | Missing | | | | | |
|  | 2 | | | | | | 2 | | | | | | 0 | | | | | | 0 | | | | | | 0 | | | | | | 0 | | | | | |
| N) At home, I drink… with most meals ^1^ | Cola/  soda | | Diet cola/soda | | | | | Fruit drink or punch | | | | | Milk | | | | Tea/coffe | | | | Juice | | | | Water | | | | | Other | | | | | Missing | |
|  | 0 | | 1 | | | | | 1 | | | | | 1 | | | | 2 | | | | 2 | | | | 4 | | | | | 0 | | | | | 0 | |
| O) At the firehouse, I drink… with most meals ^2^ | Cola/  soda | | Diet cola/soda | | | | | Fruit drink or punch | | | | | Milk | | | | Tea/coffee | | | | Juice | | | | Water | | | | | Other | | | | | Missing | |
|  | 0 | | 1 | | | | | 1 | | | | | 1 | | | | 2 | | | | 2 | | | | 4 | | | | | 0 | | | | | 0 | |

^1^ Weighted by the proportion of meals at home relative to the total number of meals per week (breakfast + lunch + dinner)

^2^ Weighted by the proportion of meals at home relative to the total number of meals per week (breakfast + lunch + dinner)
