# Supplementary material for: Modified Mediterranean Diet Score and Cardiovascular Risk in a North American Working Population
Source: PLoS One. 2014 Feb 4;9(2):e87539. doi: 10.1371/journal.pone.0087539 (PMC3913651; doi:10.1371/journal.pone.0087539)
Supplement: Method S1 — Calculation of the modified Mediterranean diet score (mMDS). (DOC) [file pone.0087539.s002.doc]

**Method S1.** Calculation of the modified Mediterranean diet score (mMDS)

Modified Mediterranean diet score (mMDS) = A + B + C + H + K + M + (D + E + I + N)*proportion of meals at home + (F + G + J + O)*proportion of meals at the firehouse
